# Association of *FTO* rs9939609 Polymorphism With Obesity and Elevated Glycemic Parameters in a Syrian Population: A Case–Control Study

**DOI:** 10.1155/jdr/1454695

**Published:** 2026-06-08

**Authors:** Lana Wehbi, Shaden Haddad

**Affiliations:** ^1^ Department of Biochemistry and Microbiology, Faculty of Pharmacy, Damascus University, Damascus, Syria, damascusuniversity.edu.sy

**Keywords:** *FTO* gene, obesity, polymorphism, rs9939609, Syria, Type 2 diabetes

## Abstract

**Background:**

Obesity is a major risk factor for Type 2 diabetes mellitus (T2DM), with substantial genetic determinants. The fat mass and obesity–associated (*FTO*) gene rs9939609 polymorphism has been consistently associated with obesity risk across diverse populations. This case–control study is aimed at investigating *FTO* rs9939609 and its associations with obesity risk and glycemic parameters in a Syrian population for the first time.

**Methods:**

A total of 97 participants (50 obese cases and 47 nonobese controls) were recruited from two university hospitals in Damascus, Syria. Among obese participants, 25 had T2DM, including 11 newly diagnosed, treatment‐naïve patients. Genotyping was performed using PCR‐RFLP, and associations were assessed using logistic and linear regression analyses.

**Results:**

The minor allele frequency was significantly higher in obese cases compared to controls (36.0% vs. 20.21%, *p* = 0.015). Under the dominant genetic model, risk allele carriers exhibited a 3.76‐fold increased obesity risk (95% CI: 1.62–8.74, *p* = 0.002). This association was preserved when the analysis was restricted to nondiabetic obese participants. No significant associations were observed between genotypes and anthropometric parameters within the obese group. Among obese nondiabetic individuals, A‐allele carriers showed significantly elevated fasting blood glucose (*p* = 0.004) and HbA1c levels (*p* = 0.034) compared to TT homozygotes; these associations remained significant after adjusting for BMI, WC, and WHR in separate models. A pooled analysis of all obese T2DM patients (*n* = 25) revealed significantly higher HbA1c in A‐allele carriers (*p* = 0.027), with exploratory subgroup analyses suggesting a stronger association in treatment‐naïve patients, though these findings require confirmation in larger studies.

**Conclusions:**

Our study provides the first evidence that *FTO* rs9939609 is significantly associated with obesity risk in the Syrian population and may contribute to early glycemic dysregulation in genetically susceptible obese individuals. Subgroup findings warrant validation in larger cohorts.

## 1. Introduction

The global burden of obesity has reached alarming proportions, presenting one of the most formidable public health challenges of the twenty‐first century. The World Health Organization (WHO) reports that approximately 16% of adults worldwide were living with obesity and 43% were classified as overweight in 2022 [1]. Parallel to this, Type 2 diabetes mellitus (T2DM) has emerged as a major health crisis with risk of developing serious complications, approximately 589 million adults living with diabetes in 2024, and projections indicating escalation to 853 million by 2050, according to the International Diabetes Federation (IDF) [2].

The Middle East and North Africa (MENA) region exhibits the highest regional diabetes prevalence worldwide [3], with meta‐analytic estimates indicating obesity and overweight prevalence of 21.2% and 33.1%, respectively, among adults [4]. Syria, situated within this high‐burden region, faces substantial challenges in addressing these metabolic epidemics.

The strong bidirectional relationship between obesity and T2DM is well documented, with obesity constituting the primary modifiable risk factor for T2DM through mechanisms involving insulin resistance, chronic inflammation, and *β*‐cell dysfunction [5].

The etiology of obesity involves complex gene–environment interactions, and genome‐wide association studies (GWASs) have identified numerous susceptibility loci. Among these, the fat mass and obesity–associated (*FTO*) gene has emerged as the first and most consistently replicated genetic determinant of common obesity [6]. In particular, the rs9939609 (T > A) variant represents the most extensively investigated single‐nucleotide polymorphism (SNP) since its landmark identification in 2007 [6]. This variant demonstrates robust associations with obesity risk across Caucasian, Asian, and other ethnic populations [7, 8].

The *FTO* gene, mapped to chromosome 16q12.2, encodes a 2‐oxoglutarate and Fe(II)‐dependent nucleic acid demethylase belonging to the AlkB family [9]. This protein catalyzes the oxidative demethylation of N6‐methyladenosine (m^6^A) modifications in RNA, representing a key epitranscriptomic regulatory mechanism, thereby regulating mRNA stability, splicing, and translation [10]. The *FTO* gene demonstrates high expression in hypothalamic nuclei involved in energy homeostasis, suggesting a central role in appetite control. Furthermore, FTO protein modulates ghrelin mRNA stability, linking it to appetite regulation and fat intake preference [11, 12]. Beyond its direct enzymatic role, the intronic region harboring obesity‐associated SNPs functions as a long‐range enhancer, modulating adjacent genes such as *IRX3*, *IRX5*, and *RPGRIP1L*, which play critical roles in adipocyte thermogenesis and energy balance [12, 13].

The minor A allele of rs9939609 elevates FTO protein levels, enhancing m^6^A demethylation of transcripts involved in lipid and glucose metabolism [10, 12]. Homozygous AA carriers exhibit approximately 1.67‐fold increased obesity risk compared to TT homozygotes, with each copy of the risk allele associated with an average body mass index (BMI) increase of ~0.4 kg/m^2^ [6].

On the other hand, the relationship between *FTO* rs9939609 and T2DM risk has been extensively investigated. Although initial studies suggested that the *FTO* polymorphism–diabetes association was primarily mediated through BMI, multiple meta‐analyses have demonstrated that rs9939609 maintains significant T2DM risk even after BMI adjustment [14, 15]. Furthermore, *FTO* gene variants have been associated with cardiovascular disease, diabetic retinopathy, and nephropathy in T2DM patients [10, 16].

*FTO* gene associations exhibit substantial interethnic variability in allele frequencies and effect estimates across diverse populations [8, 17, 18]. This heterogeneity emphasizes the importance of population‐specific genetic studies. Notably, Syria remains significantly underrepresented in genetic epidemiological research on obesity [18]. The unique genetic background of Middle Eastern populations, shaped by distinct evolutionary history and consanguinity patterns, may result in different allele frequencies and effect sizes for obesity‐associated variants.

To our knowledge, this is the first genetic association study examining the *FTO* rs9939609 polymorphism in a Syrian population. The primary objectives are (1) to determine the genotype and allele frequencies among Syrian adults with varying adiposity and glycemic status, (2) to evaluate the association between rs9939609 genotypes and obesity risk, and (3) to examine the relationship between this variant and anthropometric and glycemic parameters.

## 2. Materials and Methods

### 2.1. Study Design and Participants

This case–control study was conducted at the National University Hospital and Al‐Mwassat University Hospital in Damascus, Syria, between April 2024 and February 2025. Ethical approval was obtained from the Biomedical Research Ethics Committee at Damascus University. The study was conducted in accordance with the Declaration of Helsinki, and written informed consent was obtained from all participants prior to enrollment.

A total of 97 participants aged 40–60 years were recruited from various Syrian governorates, comprising 50 obese individuals (cases) and 47 nonobese healthy individuals (controls), frequency‐matched by age and sex. Among the obese group, 25 were diagnosed with T2DM, including 11 newly diagnosed treatment‐naïve patients. Control subjects were recruited from apparently healthy individuals attending the same hospitals for routine health check‐ups and healthy volunteers.

Inclusion criteria for cases were as follows: BMI ≥ 30 kg/m^2^ (WHO classification) [1]. T2DM diagnosis followed American Diabetes Association (ADA) criteria: fasting blood glucose (FBG) ≥ 126 mg/dL, HbA1c ≥ 6.5*%*, and/or current use of antidiabetic medications [19]. Obese nondiabetic participants had FBG < 100 mg/dL and HbA1c < 5.7*%* with no history of diabetes.

Inclusion criteria for controls were as follows: BMI 18.5–24.9 kg/m^2^, FBG < 100 mg/dL, and HbA1c < 5.7*%*, with no personal or first‐degree family history of diabetes.

Exclusion criteria included the following: thyroid dysfunction, hepatic or renal impairment, chronic pancreatitis, cardiovascular disease, anemia or hemoglobinopathies, known malignancy, pregnancy or lactation, and other forms of diabetes (T1DM, gestational diabetes, and secondary diabetes).

### 2.2. Data Collection

Demographic and clinical data were collected through face‐to‐face interviews along with medical records using a structured questionnaire. Hypertension was defined as current use of antihypertensive medications. Smoking status was recorded as current smoker or nonsmoker. Physical activity was categorized as inactive (< 30 min walking/day, sedentary occupation), low (30–60 min walking/day), or sufficient (> 60 min walking/day or moderate‐to‐vigorous occupational activity). Current antidiabetic medications were recorded for T2DM patients.

### 2.3. Anthropometric Measurements

Body weight was measured using a calibrated electronic scale with participants wearing light clothing and without shoes. Height was measured using a stadiometer to the nearest 0.1 cm. BMI was calculated as *w* *e* *i* *g* *h* *t* (*k* *g*)/*h* *e* *i* *g* *h* *t* ^2^ (*m* ^2^). Waist circumference (WC) was measured at the midpoint between the lower rib and iliac crest, and hip circumference (HC) was measured at the widest portion of the buttocks using a nonstretchable flexible tape; waist‐to‐hip ratio (WHR) was calculated accordingly.

### 2.4. Biochemical Analyses

Venous blood samples were collected in the morning after overnight fasting (8–10 h). Serum was separated for FBG measurement using the hexokinase enzymatic method (mg/dL). A 2 mL aliquot was collected in EDTA tubes for HbA1c quantification using the immunoturbidimetric method (%) and genomic DNA extraction. Both biochemical analyses were performed on a Cobas c 501 analyzer (Roche Diagnostics, Germany).

### 2.5. DNA Extraction and Genotyping

Laboratory procedures were conducted at the Molecular Biology Laboratory of the National University Hospital and the Biotherapeutic Research Center at Damascus University. Genomic DNA was extracted from 300 *μ*L of whole blood using the FlexiGene DNA Kit (Qiagen, Germany) according to the manufacturer′s protocol. DNA concentration and purity were assessed using a NanoDrop ND‐1000 spectrophotometer (Thermo Fisher Scientific, United States); all samples met quality criteria with A260/280 ratios within 1.7–2.0. Extracted DNA was stored at −20°C until analysis.

The *FTO* rs9939609 polymorphism was genotyped using polymerase chain reaction–restriction fragment length polymorphism (PCR‐RFLP). Primers were adapted from López‐Bermejo et al. [20] to amplify the *FTO* gene region containing rs9939609 and introduce a recognition site for the restriction enzyme:

• Forward: 5 ^′^‐AACTGGCTCTTGAATGAAATAGGATTCAGA‐3 ^′^
• Reverse: 5 ^′^‐AGAGTAACAGAGACTATCCAAGTGCAGTAC‐3 ^′^

PCR amplification was performed in a 25 *μ*L reaction volume containing 1.5–2 *μ*L genomic DNA template (0.2–0.5 *μ*g), 0.625 *μ*L of each primer (10 pmol/*μ*L), 0.25 *μ*L MgCl₂ (50 mM), 12.5 *μ*L of 2X Taq Master Mix (Vivantis, Malaysia), and nuclease‐free water. Thermal cycling conditions (Mastercycler Nexus GSX1, Eppendorf, Germany) were initial denaturation at 94°C for 5 min; 35 cycles of 94°C for 30 s, 61°C for 30 s, and 72°C for 60 s; and final extension at 72°C for 10 min. The expected product size was 182 bp, verified by electrophoresis on 2.5% agarose gel stained with ethidium bromide.

Restriction digestion was performed using ScaI‐HF enzyme (New England Biolabs, United States). The A allele contains a ScaI recognition site, generating fragments of 154 and 28 bp upon cleavage, while the T allele remains uncut (182 bp). Approximately 1 *μ*g of PCR product was digested with 7.5–10 units of ScaI‐HF in a 20 *μ*L reaction volume at 37°C for 1 h. Digestion products were separated on 3.5% agarose gel at 100 V for 40 min and visualized under UV transillumination. For quality control, 5% of samples were randomly selected for repeat genotyping, achieving 100% concordance.

### 2.6. Statistical Analysis

Statistical analyses were performed using SPSS Version 26.0 (IBM Corp., Armonk, New York, United States). Normality of continuous variables was assessed using the Kolmogorov–Smirnov and Shapiro–Wilk tests. As data followed a normal distribution, continuous variables were compared between groups using the independent samples *t*‐test and presented as *m* *e* *a* *n* ± *s* *t* *a* *n* *d* *a* *r* *d* *d* *e* *v* *i* *a* *t* *i* *o* *n* (SD). Variables that did not meet normality assumptions were assessed using the Mann–Whitney *U* test. Categorical variables, including genotype and allele frequencies, were compared using the chi‐square test and presented as frequencies and percentages.

The Hardy–Weinberg equilibrium (HWE) was evaluated in the control group using the chi‐square goodness‐of‐fit test.

Due to the low frequency of the AA genotype, the dominant genetic model (TA + AA vs. TT) was used for association analyses. Odds ratios (OR) with 95% confidence intervals (CIs) were calculated using logistic regression. The association between *FTO* rs9939609 genotypes and anthropometric/glycemic parameters among obese subjects was assessed using an independent *t*‐test and linear regression.

Due to the limited sample size in the treatment‐naïve T2DM subgroup (*n* = 11), genotype–glycemic associations in this group were assessed using a *t*‐test only to avoid model overfitting. A two‐tailed *p* *v* *a* *l* *u* *e* < 0.05 was considered statistically significant.

## 3. Results

### 3.1. Baseline Characteristics

A total of 97 participants were enrolled, comprising 50 obese cases and 47 nonobese controls. The two groups were well matched for age and sex distribution. As expected, obese individuals exhibited significantly higher BMI, WC, HC, and WHR compared to controls (all *p* < 0.001). Among the obese group, 50% had T2DM, and the prevalence of hypertension and physical inactivity was significantly higher in cases. Detailed characteristics are presented in Table 1.

**Table 1:** Demographic, anthropometric, and clinical characteristics of obese cases and nonobese controls.

**Continuous variables**	Cases (*n*: 50)	Controls (*n*: 47)	*p*value
Age (years)	51 ± 4.66	50.89 ± 5.82	0.921
Weight (kg)	101.61 ± 17.36	60.48 ± 6.75	< 0.001
Height (cm)	165.46 ± 10.52	168.38 ± 8.44	0.136
BMI (kg/m^2^)	36.96 ± 4.32	21.3 ± 1.37	< 0.001
Waist circumference (cm)	116.96 ± 10.06	79.06 ± 5.11	< 0.001
Hip circumference (cm)	121.26 ± 9.78	93.7 ± 5.41	< 0.001
WHR	0.97 ± 0.07	0.84 ± 0.05	< 0.001
FBG (mg/dL)			
In obese individuals without T2DM	90.56 ± 4.03	87.21 ± 5.97	0.014
In obese individuals with T2DM	162.28 ± 30.25	87.21 ± 5.97	< 0.001
HbA1c (%)			
In obese individuals without T2DM	5.52 ± 0.36	5.18 ± 0.29	< 0.001
In obese individuals with T2DM	8.62 ± 1.4	5.18 ± 0.29	< 0.001
**Categorical variables**			
Male:female ratio	26:24	24:23	
Diabetes, *n* (%)	25 (50.0)	0	< 0.001
Hypertension, *n* (%)	18 (36.0)	4 (8.51)	0.001
Smoking, *n* (%)	22 (44.0)	20 (42.55)	0.886
Physical activity level			
Physically inactive	15 (30.0)	3 (6.38)	< 0.001
Physical activity level low	25 (50.0)	11 (23.4)	
Physical activity level sufficient	10 (20.0)	33 (70.21)	

Abbreviations: BMI, body mass index; FBG, fasting blood glucose; HbA1c, glycated hemoglobin; T2DM, Type 2 diabetes mellitus; WHR, waist‐to‐hip ratio.

### 3.2. PCR‐RFLP Genotyping

**Figure 1:**
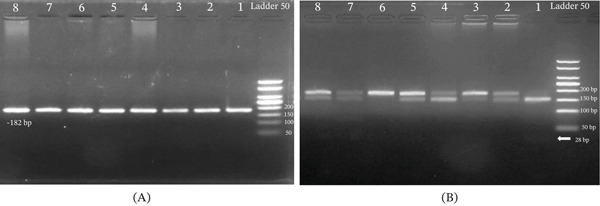
Agarose gel electrophoresis of the *FTO* rs9939609 variant. (A) PCR products showing 182 bp amplicon (Lanes 1–8) on 2.5% agarose gel. (B) ScaI‐digested PCR products on 3.5% agarose gel showing different genotypes: Lane 1, AA genotype; Lanes 2, 3, 4, 5, 7, and 8, TA genotype; and Lane 6, TT genotype. A 50 bp DNA ladder was used in both panels. The 28 bp fragment is faintly visible due to its small size.

The *FTO* rs9939609 polymorphism was successfully genotyped in all participants using PCR‐RFLP analysis. Agarose gel electrophoresis revealed distinct banding patterns corresponding to the three genotypes: TT (182 bp), AA (154 + 28 bp), and TA (182 + 154 + 28 bp), as shown in Figure 1.

### 3.3. Genotype and Allele Frequencies

The genotype distribution in controls was consistent with HWE (*p* = 0.405). The overall genotype distribution differed significantly between groups (*p* = 0.007), with the TA genotype notably more frequent in obese cases (Table 2).

**Table 2:** Genotype and allele frequency distribution of *FTO* rs9939609 polymorphism and association with obesity risk.

*FTO* rs9939609	Cases (*n*: 50)	Controls (*n*: 47)	*χ* ^2^	*p*value	OR (95% CI)
Genotypes					
TT	15 (30.0)	29 (61.7)			
TA	34 (68.0)	17 (36.17)	10	**0.007**	—
AA	1 (2.0)	1 (2.13)			
Dominant model					
TT	15 (30.0)	29 (61.7)	—	—	1.00 (Ref.)
TA + AA	35 (70.0)	18 (38.3)	10	**0.002**	**3.76 (1.62–8.74)**
Alleles					
T	64 (64.0)	75 (79.79)	—	—	1.00 (Ref.)
A	36 (36.0)	19 (20.21)	6	**0.015**	**2.22 (1.16–4.26)**

*Note:* Genotype distribution in controls was consistent with the Hardy–Weinberg equilibrium (*χ* ^2^ = 0.692, *p* = 0.405). Bold values indicate statistical significance (*p*<0.05)

Abbreviations: *χ* ^2^, chi‐square test; CI, confidence interval; OR, odds ratio.

Due to low AA genotype frequency, the dominant model (TA + AA vs. TT) was used. Individuals carrying at least one copy of the A allele exhibited a 3.76‐fold increased obesity risk compared to those with the TT genotype. The minor allele frequency was significantly higher in cases than controls (36.0% vs. 20.21%, *p* = 0.015).

### 3.4. Multivariable Logistic Regression Analysis

To assess whether the association between *FTO* rs9939609 and obesity was independent of potential covariates, multivariable logistic regression analyses were performed under the dominant model (Table 3). The association persisted after adjustment for age and sex, and was stronger after separate adjustment for physical activity assessed with questionnaires. To address potential confounding from T2DM comorbidity within the obese group (50%), a sensitivity analysis was performed restricted to nondiabetic obese participants (*n* = 25) versus controls (*n* = 47), which preserved the association across all models, confirming the robustness of the *FTO*–obesity association in the Syrian population.

**Table 3:** Multivariable logistic regression analysis for the association between *FTO* rs9939609 and obesity.

Groups	Dominant model	Unadjusted model		Model 1		Model 2	
		*p*value	OR (95% CI)	*p*value	OR (95% CI)	*p*value	OR (95% CI)
Obese (*n* = 50), controls (*n* = 47)	TT	—	1	—	1	—	1
	TA + AA	0.002	3.76 (1.62–8.74)	0.002	3.76 (1.62–8.75)	0.002	5.28 (1.85–15.07)

Obese nondiabetic (*n* = 25), controls (*n* = 47)	TT	—	1	—	1	—	1
	TA + AA	0.003	5.1 (1.72–15.18)	0.003	5.18 (1.73–15.52)	0.004	5.91 (1.76–19.93)

*Note:* Obesity is classified as “nonobese = 0” and “obese = 1.” Model 1: Adjusted for age and sex. Model 2: Adjusted for physical activity level. The “inactive” and “low” activity levels were combined into one category as “insufficient physical activity.” The reference category for genotype is “TT genotype,” and for physical activity, it is “Physical activity level sufficient.”

Abbreviations: CI, confidence interval; OR, odds ratio.

### 3.5. Association With Anthropometric and Glycemic Parameters

Glycemic and anthropometric parameters by genotype within the obese cohort and its subgroups are presented in Table 4. Risk allele carriers exhibited a borderline higher WHR compared to TT homozygotes (*p* = 0.028), although this difference did not retain statistical significance after adjustment for age and sex (*p* = 0.053). No significant differences were observed between genotypes and the other anthropometric parameters (all *p* > 0.05).

**Table 4:** Association of *FTO* rs9939609 genotypes with anthropometric and glycemic parameters among obese subjects.

General criteria	TT (*n*: 15)	TA + AA (*n*: 35)	*p*value
M:F ratio	6: 9	20:15	0.175
Age (years)	51.36 ± 5.3	50.94 ± 4.5	0.783
Indicators of obesity			
Weight (kg)	100.95 ± 19.74	101.89 ± 16.54	0.863
BMI (kg/m^2^)	37.9 ± 4.95	36.55 ± 4.03	0.317
Waist circumference (cm)	116.27 ± 12.12	117.26 ± 9.23	0.753
Hip circumference (cm)	124.93 ± 11.93	119.51 ± 8.04	0.123
WHR	0.93 ± 0.07	0.98 ± 0.07	0.028/0.053^a^
Glycemic parameters			
Obese without T2DM (*n* = 25)	TT (*n*: 6)	TA + AA (*n*: 19)	
FBG (mg/dL)	86.67 ± 3.56	91.79 ± 3.39	0.004
HbA1c (%)	5.26 ± 0.51	5.61 ± 0.27	0.034
All obese T2DM patients (*n* = 25)	TT (n: 9)	TA + AA (n: 16)	
FBG (mg/dL)	148.67 ± 23.92	169.94 ± 31.39	0.065
HbA1c (%)	7.83 ± 1.38	9.06 ± 1.24	0.027
Obese with T2DM—receiving antidiabetic medications (*n* = 14)	TT (*n*: 5)	TA + AA (*n*: 9)	
FBG (mg/dL)	159 ± 28.31	164.78 ± 27.58	0.716
HbA1c (%)	8.62 ± 1.42	8.5 ± 1.06	0.855
Obese with T2DM—newly diagnosed/naive (*n* = 11)	TT (*n*: 4)	TA + AA (*n*: 7)	
FBG (mg/dL)	135.75 ± 7.5	176.57 ± 36.86	0.026
HbA1c (%)	6.83 ± 0.15	9.79 ± 1.12	0.001

Abbreviations: BMI, body mass index; FBG, fasting blood glucose; HbA1c, glycated hemoglobin; T2DM, Type 2 diabetes mellitus; WHR, waist‐to‐hip ratio.

^a^ *p* value adjusted for age and sex by linear regression analysis.

Among obese nondiabetic individuals (*n* = 25), A‐allele carriers showed significantly elevated FBG (*p* = 0.004) and HbA1c (*p* = 0.034) compared to TT homozygotes. Among all obese T2DM patients (*n* = 25), pooled analysis revealed that A‐allele carriers exhibited significantly higher HbA1c (*p* = 0.027), with a similar trend for FBG (*p* = 0.065). Subsequent stratification by treatment status revealed that this association was driven by newly diagnosed, treatment‐naïve T2DM patients (*n* = 11), where A‐allele carriers showed significantly higher FBG (*p* = 0.026) and HbA1c (*p* = 0.001), while no significant associations were detected in patients receiving antidiabetic medications (*n* = 14; FBG: *p* = 0.716; HbA1c: *p* = 0.855).

No statistically significant associations were observed between *FTO* rs9939609 genotypes and any of the measured parameters in the control group (data not shown in tables).

Linear regression analysis confirmed that the associations between *FTO* rs9939609 and glycemic parameters in obese nondiabetic individuals remained significant after adjustment for age, sex, and adiposity measures (BMI, WC, or WHR) in three separate models (Table 5).

**Table 5:** Linear regression analysis for the association of *FTO* rs9939609 with glycemic parameters among obese nondiabetic subjects.

Parameter	Cases	Model	*B*(SE)	95% CI	*p*value
FBG (mg/dL)	Obese nondiabetic (*n* = 25)	A	5.28 (1.62)	1.9–8.67	0.004
		B	5.35 (1.65)	1.9–8.80	0.004
		C	5.63 (1.66)	2.17–9.09	0.003

HbA1c (%)	Obese nondiabetic (*n* = 25)	A	0.41 (0.18)	0.04–0.78	0.031
		B	0.42 (0.18)	0.05–0.79	0.030
		C	0.44 (0.18)	0.06–0.81	0.025

*Note:* Model A: Adjusted for age, sex, and body mass index (BMI). Model B: Adjusted for age, gender, and waist circumference (WC). Model C: Adjusted for age, gender, and waist‐to‐hip ratio (WHR).

Abbreviations: *B*, unstandardized coefficient; CI, confidence interval; FBG, fasting blood glucose; HbA1c, glycated hemoglobin; SE, standard error.

## 4. Discussion

Obesity is a major global health concern linked to metabolic syndrome, Type 2 diabetes, and cardiovascular disease. The genetic architecture underlying obesity susceptibility varies across ethnic groups, and Middle Eastern populations remain underrepresented in obesity genetics research. This study represents the first investigation of the *FTO* rs9939609 polymorphism and its association with obesity susceptibility in the Syrian Arab population.

Our study revealed a strong association between the *FTO* rs9939609 polymorphism and obesity risk in the studied Syrian population, with the heterozygous TA genotype being the primary driver. Carriers of the A allele exhibited an OR of 3.76 for obesity compared to TT homozygotes (95% CI: 1.62–8.74; *p* = 0.002; Table 3), and this association persisted after adjustment for age and sex, was stronger after separate adjustment for self‐reported physical activity. The persistence of this association in the sensitivity analysis restricted to nondiabetic obese participants supports that the observed effect reflects a genuine *FTO* rs9939609–obesity association rather than selection bias attributable to T2DM comorbidity.

The strengthening of the OR after adjustment for physical activity suggests that this environmental factor may have been masking part of the true genetic effect, reinforcing the hypothesis of gene–environment interaction and partially explaining why some control participants carrying the A allele with sufficient physical activity did not develop obesity. However, more precise characterization of these gene–environment interactions requires dedicated studies, particularly given the self‐reported nature of the questionnaire and the inability to incorporate dietary intake into the analysis.

Our findings are consistent with the growing body of literature establishing the role of *FTO* rs9939609 in increasing obesity risk, with large‐scale meta‐analyses reporting per‐allele ORs ranging from 1.20 to 1.31 across multiple populations [7, 17]. However, the ORs observed in our cohort (OR = 3.76 in the overall obese group and OR = 5.18 in nondiabetic obese participants under the dominant model) appear higher than those typically reported. Within the Arab region, single‐population studies have reported more modest effect sizes—1.46‐fold in Tunisia [21] and 1.47‐fold in Kuwait [22]—whereas studies in non‐Arab Asian populations have approached our estimates, with reported ORs of 2.61 in China [23] and 3.02 in Pakistan [24]. In contrast, null findings have been reported in the Turkish population [25], highlighting the well‐documented variability of this polymorphism′s effect across ethnic backgrounds [8].

Our higher effect estimates likely reflect the convergence of several factors. Foremost are the stringent control selection criteria, which excluded any individual with obesity, T2DM, or a family history of diabetes—an approach that contrasts with studies whose less rigorously screened controls may attenuate the apparent effect size through higher background A‐allele frequencies. Additionally, the modest sample size may have contributed to the magnitude of the estimated effect, as reflected by the relatively wide CIs in the nondiabetic obese subgroup (*n* = 25). The ethnic diversity of the Syrian population may also yield a distinctive genetic profile differing from more homogeneous populations. Nevertheless, the persistence of statistical significance across all adjustment models and both obese subgroups supports the presence of a genuine biological signal that warrants validation in larger cohorts.

The strength of this association can be understood in light of FTO′s multitissue mechanisms of action. As an epitranscriptomic regulator, FTO dynamically modulates m^6^A levels across a broad spectrum of mRNA transcripts involved in metabolic processes, resulting in coordinated effects across multiple tissues simultaneously [10]. FTO promotes energy intake through impaired hypothalamic satiety signaling and increased appetite [11, 26], increased hepatic de novo lipogenesis [27], and altered adipocyte differentiation through alternative splicing of *RUNX1T1* [28]. Concurrently, FTO suppresses energy expenditure by inhibiting fatty acid oxidation [27] and reducing adipocyte browning and thermogenesis through long‐range *cis*‐regulation of neighboring genes (*IRX3* and *IRX5*) [13]. Collectively, these axes constitute an integrated metabolic network that drives sustained weight gain through chronic energy imbalance—effects that, given the persistent nature of this polymorphism′s regulatory action, accumulate over time.

The Syrian population in our study showed a notably low AA genotype frequency, and the control A‐allele frequency (20.21%) was lower than that reported in the broader Middle East (38.4%), European (40.7%), and African (47.9%) populations while closer to East Asian (15.5%) and South Asian (31.5%) frequencies [29]. In contrast, the A‐allele frequency rose to 36% in the overall obese group, approaching values reported in Arab, European, and South Asian studies. This intermediate pattern is consistent with the well‐documented geographic and ethnic variability in this polymorphism [30, 31] and likely reflects the Syrian Levant′s geographic and historical position at the crossroads of multiple ancestral populations [32].

Within our obese cohort, no significant associations were observed between *FTO* rs9939609 genotypes and anthropometric parameters. Although a borderline higher WHR was observed in A‐allele carriers, this difference did not retain statistical significance after adjustment for age and sex. Similar null findings have been reported in Turkish [25] and Thai populations [33]. Ağagündüz and Gezmen‐Karadaǧ [34] suggested that *FTO* rs9939609 influences overall fat accumulation rather than abdominal fat distribution, potentially explaining the lack of association with WC, HC, or WHR.

This null finding may also reflect methodological considerations. Studies reporting positive associations between *FTO* rs9939609 and anthropometric measures typically analyzed entire cohorts spanning the full BMI spectrum, whereas our within‐group analysis was restricted to obese individuals, where a ceiling effect may attenuate genetic associations. Furthermore, the low AA genotype frequency (2%) limited statistical power to detect the modest per‐allele effect of ~0.3–0.4 kg/m^2^ reported in the literature [6]. Sex‐stratified analyses may also reveal differential associations, given the distinct fat distribution patterns between males and females and previously reported sex‐specific *FTO* rs9939609 effects [35, 36]; however, our limited sample size precluded such stratification.

Carriers of the A allele exhibited a 5.28–5.63 mg/dL increase in FBG and a 0.41%–0.44% increase in HbA1c compared to TT homozygotes among nondiabetic obese individuals, with these associations remaining significant after adjustment for BMI, WC, and WHR (Table 5). The independence of this effect from adiposity measures suggests that rs9939609 may contribute to glucose dysregulation in obese individuals prior to reaching the clinical threshold for T2DM diagnosis. We acknowledge, however, that direct measurement of fasting insulin and HOMA‐IR would have allowed more precise characterization of insulin resistance, reinforcing the need for future studies incorporating these parameters.

This association can be understood through a sequential mechanism: The A allele increases obesity risk and, subsequently, exacerbates metabolic dysregulation through interconnected pathways—interference with insulin signal transduction, chronic inflammation impairing *β*‐cell function, and dysregulation of hepatic gluconeogenesis [10, 37]. The A allele likely deepens this disturbance, accelerating *β*‐cell functional decline and driving carriers toward the clinical threshold for T2DM diagnosis more rapidly than noncarriers.

Our findings align with several studies reporting associations between *FTO* rs9939609 and glycemic dysregulation across diverse populations. Shahid et al. demonstrated a significant association between the A allele and elevated FBG in obese Pakistani individuals [38], while Saber‐Ayad et al. reported elevated FBG in AA carriers within an Emirati cohort [39]. Binh et al. [40] further showed *FTO* rs9939609 as a predictor of future T2DM, while Song et al. [41] showed that FBG mediated 45.76% of the rs9939609–metabolic syndrome association.

Of particular relevance, recent work by Fragoso‐Bargas et al. in the Mexican population demonstrated that rs9939609‐A is associated with elevated FBG and HbA1c, with associations persisting after adjustment for BMI, WC, and WHR [42]—a methodological convergence with our analytical approach (Table 5). The consistency of *FTO* rs9939609–glycemic associations across populations often underrepresented in genome‐wide studies—including the Syrian and Mexican populations—and across Pakistani, Emirati, Vietnamese, and Chinese cohorts reinforces the notion that rs9939609 confers a reproducible effect on glucose dysregulation that transcends ethnic boundaries.

To further explore the relationship between rs9939609 and glycemic dysregulation among diabetic patients, we performed a pooled analysis of all obese T2DM patients (*n* = 25). A‐allele carriers exhibited significantly higher HbA1c levels (*p* = 0.027) with a trend toward elevated FBG (*p* = 0.065) compared to TT homozygotes (Table 4). The persistence of a significant HbA1c difference across the entire T2DM cohort—including patients receiving pharmacological treatment—supports that rs9939609 is associated with glycemic dysregulation across the spectrum of T2DM.

Stratification by treatment status further revealed that the genotype–glycemic association was driven by the newly diagnosed, treatment‐naïve subgroup (*n* = 11), where A‐allele carriers exhibited markedly elevated HbA1c at diagnosis (*p* = 0.001) and significantly higher FBG (*p* = 0.026) compared to TT homozygotes. This finding suggests an association between A‐allele carriage and more pronounced glycemic dysregulation at T2DM diagnosis. Given the small subgroup size, however, this finding should be considered exploratory and requires confirmation in larger cohorts.

In contrast, no significant genotype–glycemic association was detected in the medicated subgroup (*n* = 14), although several methodological constraints likely contributed to this null finding. This subgroup was clinically heterogeneous in disease duration and pharmacological regimens (varying in number of agents, dosages, and administration frequencies); the small subgroup size precluded fine‐grained stratification by drug class or treatment regimen. Combined with the very low AA genotype frequency (~2%), these factors collectively limited statistical power. Notably, recent evidence from Fragoso‐Bargas et al. [43] demonstrated that rs9939609‐A remains associated with poorer glycemic control in a Mexican T2DM cohort under pharmacological treatment, while Hussain et al. reported an FTO–glycemic association in Pakistani T2DM patients confined to AA homozygotes [16]. These observations suggest that the absence of association in our medicated subgroup likely reflects methodological constraints rather than evidence against a true *FTO* effect, underscoring the need for dedicated studies in larger, well‐characterized cohorts.

Several limitations should be acknowledged. First, the modest sample size (*n* = 97) and particularly the small treatment‐naïve T2DM subgroup (*n* = 11) may have limited statistical power; subgroup findings should, therefore, be considered exploratory. Second, the very low frequency of the AA genotype (~2%) precluded full dose–response characterization. Third, we did not measure fasting insulin or HOMA‐IR for comprehensive insulin resistance assessment, and physical activity was assessed through a self‐reported questionnaire. Fourth, the cross‐sectional design cannot establish temporal relationships. Future prospective studies in larger Syrian cohorts, incorporating insulin resistance assessment, objective lifestyle measurements, dietary assessment, and additional *FTO* variants, are warranted.

## 5. Conclusions

This study provides the first evidence that *FTO* rs9939609 is significantly associated with obesity risk in the Syrian population, with risk allele carriers showing a 3.76‐fold higher risk of obesity. The *FTO* rs9939609 variant was also associated with elevated glycemic parameters in obese nondiabetic individuals and across the spectrum of obese T2DM patients, suggesting that this variant may contribute to early glycemic dysregulation in genetically susceptible individuals. However, findings in subgroup analyses, particularly in the small treatment‐naïve subgroup, should be considered exploratory and warrant validation in larger cohorts. These findings add valuable data from an understudied MENA population to the global understanding of *FTO* genetics. Future prospective studies with larger sample sizes, comprehensive assessment of insulin resistance, and examination of gene–environment interactions are warranted.

**Nomenclature**
ADA: American Diabetes Association
BMI: body mass index
CI: confidence interval
DNA: deoxyribonucleic acid
EDTA: ethylenediaminetetraacetic acid
FBG: fasting blood glucose
*FTO*
: fat mass and obesity–associated (gene)
GWAS: genome‐wide association study
HbA1c: glycated hemoglobin
HC: hip circumference
HOMA‐IR: homeostatic model assessment of insulin resistance
HWE: Hardy–Weinberg equilibrium
IDF: International Diabetes Federation
m^6^A: N6‐methyladenosine
MENA: Middle East and North Africa
mRNA: messenger ribonucleic acid
OR: odds ratio
PCR‐RFLP: polymerase chain reaction–restriction fragment length polymorphism
RNA: ribonucleic acid
SD: standard deviation
SE: standard error
SNP: single‐nucleotide polymorphism
SPSS: Statistical Package for the Social Sciences
T1DM: Type 1 diabetes mellitus
T2DM: Type 2 diabetes mellitus
UV: ultraviolet
WC: waist circumference
WHO: World Health Organization
WHR: waist‐to‐hip ratio

## Author Contributions

L.W.: conceptualization, methodology, investigation, data curation, formal analysis, and writing—original draft, review, and editing. S.H.: conceptualization, methodology, supervision, validation, and writing—review and editing.

## Funding

No funding was received for this manuscript.

## Disclosure

All authors have read and agreed to the published version of the manuscript.

## Ethics Statement

This study was conducted in accordance with the Declaration of Helsinki. The protocol was approved by the Biomedical Research Ethics Committee at Damascus University (Approval No. PH‐210224‐209; Date: February 21, 2024). Written informed consent was obtained from all participants prior to enrollment in the study.

## Conflicts of Interest

The authors declare no conflicts of interest.

## Data Availability

The datasets used and/or analyzed during the current study are available from the corresponding author upon reasonable request.
